# The leaf angle distribution of natural plant populations: assessing the canopy with a novel software tool

**DOI:** 10.1186/s13007-015-0052-z

**Published:** 2015-02-26

**Authors:** Mark Müller-Linow, Francisco Pinto-Espinosa, Hanno Scharr, Uwe Rascher

**Affiliations:** Institute of Bio and Geosciences, IBG-2: Plant Sciences, Forschungszentrum Jülich GmbH, Wilhelm-Johnen-Str, Jülich, 52425 Germany

**Keywords:** Stereo imaging, 3-d reconstruction, Leaf segmentation, Leaf modeling, Canopy, Structural canopy straits, Leaf angle, Leaf angle distribution

## Abstract

**Background:**

Three-dimensional canopies form complex architectures with temporally and spatially changing leaf orientations. Variations in canopy structure are linked to canopy function and they occur within the scope of genetic variability as well as a reaction to environmental factors like light, water and nutrient supply, and stress. An important key measure to characterize these structural properties is the leaf angle distribution, which in turn requires knowledge on the 3-dimensional single leaf surface. Despite a large number of 3-d sensors and methods only a few systems are applicable for fast and routine measurements in plants and natural canopies. A suitable approach is stereo imaging, which combines depth and color information that allows for easy segmentation of green leaf material and the extraction of plant traits, such as leaf angle distribution.

**Results:**

We developed a software package, which provides tools for the quantification of leaf surface properties within natural canopies via 3-d reconstruction from stereo images. Our approach includes a semi-automatic selection process of single leaves and different modes of surface characterization via polygon smoothing or surface model fitting. Based on the resulting surface meshes leaf angle statistics are computed on the whole-leaf level or from local derivations. We include a case study to demonstrate the functionality of our software. 48 images of small sugar beet populations (4 varieties) have been analyzed on the base of their leaf angle distribution in order to investigate seasonal, genotypic and fertilization effects on leaf angle distributions. We could show that leaf angle distributions change during the course of the season with all varieties having a comparable development. Additionally, different varieties had different leaf angle orientation that could be separated in principle component analysis. In contrast nitrogen treatment had no effect on leaf angles.

**Conclusions:**

We show that a stereo imaging setup together with the appropriate image processing tools is capable of retrieving the geometric leaf surface properties of plants and canopies. Our software package provides whole-leaf statistics but also a local estimation of leaf angles, which may have great potential to better understand and quantify structural canopy traits for guided breeding and optimized crop management.

## Introduction

Canopies of plant populations are featured by functional designs with a complex arrangement of leaves and stems, which are subject to temporal and spatial fluctuations on various scales. The potential of leaf display is defined by the genetic framework, which sets up the range for development and environmental responses. Different temporal scales are involved: (i) leaves and canopy element are passively moved by wind causing a highly fluctuating light environment within the canopy [1]; (ii) on the diurnal time scale several leaf movement and sun tracking strategies were described aiming for either optimal light interception or avoidance of high light conditions [2-5]; (iii) growth and developmental processes change and alter canopy structure during the seasonal cycle and as a reaction to environmental stresses [6,7]. During their seasonal development most plants display strong morphological changes, which depend on the availability of resources and on the fluctuation of abiotic factors. Structural properties which are altered on the diurnal and seasonal basis may affect the efficiency of light interception within the canopy and thus may influence canopy light use efficiency [5,8,9]. Regarding the spatial scales structural shaping and adaptive reactions do not happen uniformly but strongly depend on the vertical and horizontal distribution of stems and leaves. Light availability becomes increasingly limited and fluctuating in the lower canopy layers and plants may adapt to this by layer-specific distributions of leaf orientations. These structure-function relations have recently being put in the focus of breeding strategies as potential yield improvements using biochemical optimization of photosynthesis have reached an optimum, while structural optimization may still pose some potential to improve canopy light use efficiency [10].

One of the geometric key measures, which has been used most commonly, is the leaf area index (LAI), which simply relates plant surface to soil surface. While this parameter has been intensively studied (potential and limitations of LAI measurements are discussed in [11-13]), the LAI does not provide any information on leaf architecture or the distribution of leaf orientations. Leaf orientation can be greatly affected by environmental influences like drought, which makes this an interesting trait for breeders when comparing drought stress tolerance between cultivars. Most of the available studies are dealing with single plants [14], are implemented under lab conditions [15,16] or use virtual plant models [17-19]. Others use labor-intensive manual methods and thus are based on a limited sample size [20]. Especially when dealing with lab-based applications and single plants, reconstructions of the entire canopy may be accomplished. Only a few studies focus on a quantitative assessment of leaf orientations of the outer canopy layers of plants grown under field conditions [21-23]. Despite the importance of measuring structural traits such as the leaf angle distribution, there is currently no method available, that (i) can easily be used under field conditions, (ii) works on changing canopies that are often moved by wind or (iii) delivers a high sample size of a representative number of leaves within a canopy with high spatial resolution. All these requirements are essential for knowledge-guided crop breeding [24,25], where the rating by visual judgment is still the most common method. Despite the variety of sensor-supported methods, which entered this field of phenotyping in the recent years with a high potential to assist the labor-intensive work and to replace some of the subjective ratings with automatic registration routines, only a minor method and knowledge transfer has taken place [26]. For the non-manual estimation of leaf angles several methodical approaches are available which include structured light approaches [14], stereo imaging [7,27] and laser scanning techniques [28-30]. A suitable 3-d system for rapid plant phenotyping should facilitate easy segmentation of leaves and be applicable under field conditions, thus should yield stable results also under windy conditions.

With this communication we present a fundamental step forward in using stereo camera approaches to quantify the outer canopy layer of different experimental plant systems. Our method was first developed by Biskup et al. [27] and then further refined and applied in Rascher et al. [9]. This approach uses a set of two consumer cameras that are mounted a few meters top-of-canopy. Stereo images are taken without major constraints making it an easy to use field set-up. A pipeline of computer routines later allows the calculation of depth maps, allows for the segmentation of leaf sections and the estimation of leaf orientations. However, this first scientific version required advanced computer knowledge to handle the processing routines and the system only fitted a planar leaf model and did not allow for natural leaf geometry. Herewith, we introduce a fundamentally revised and in many aspects extend version of the system of Biskup et al. [27]. Software routines were re-programmed and equipped with graphical user interfaces within the MatLab environment now being substantially more robust. A user guided semi-automated leaf segmentation routine was added and the 3-d images are now fitted with realistic and flexible leaf models allowing for a local derivation of leaf orientations. To cope with the susceptibility of field-acquired data to noise we included several filters and alternative processing paths which may be selected according to the particular problem. Different levels of user interaction are implemented reaching from default paths up to precise adjustment of parameters.

## Implementation

### Setup of a stereo camera system

**Figure 1:**
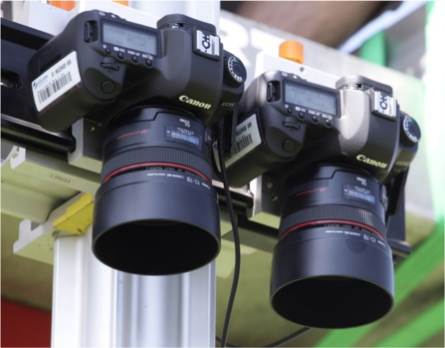
**Stereo rig.** Two cameras are fixed with a profile system and slightly turned to each other to capture the same scene. A remote-control release allows for synchronous triggering. In this setup the baseline was adjusted to *b*≈200 *m* *m*.

Our software package has been developed to derive 3-d surface models and leaf surface parameters from stereo images of plants and plant canopies taken in a nadir perspective using ordinary cameras and lenses with a fixed focal length *f*. Such a setup is easy to handle and needs only a few additional components like a calibration pattern (for target calibration) and optionally an inclinometer and a compass to correct the direction and misalignment of the stereo rig. Figure 1 shows our example of the stereo rig. The two cameras need to be aligned in a fixed geometry (two clamps at the rigid metal profile in Figure 1). This setup is calibrated and must not be changed during image acquisition. This means also that all camera internal automation, especially auto-focus, need to be turned off. If applied on non-rigid objects like a moving canopy simultaneous exposure is of crucial importance and therefore cameras should be triggered via a remote-control release. To find the best depth resolution and depth of focus we provide a tool which helps to estimate the distances between cameras (which is the baseline *b*) and the distance between stereo rig and plants.

### Software

**Figure 2:**
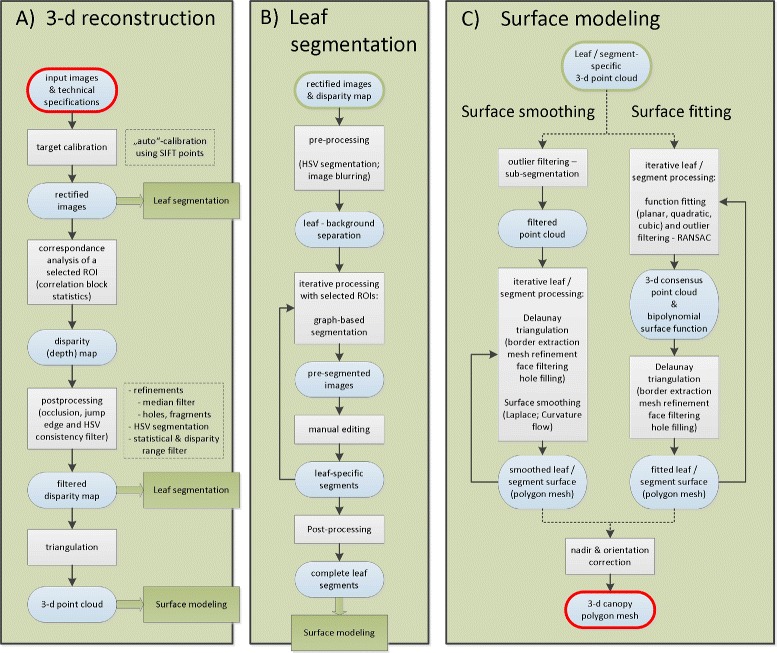
**Box diagram of module 1-3.** General outline of the leaf angle processing toolbox: The structure of this toolbox comprises 3 basic modules **(A-C)**, all of them controlled by individual graphical user interfaces (these GUIs are depicted in Figures 3, 4, and 5). Optional GUI-supported tools for HSV segmentation, stereo rig settings and result summary are not illustrated here. Module 3 comprises two alternative processing paths (left: via surface smoothing; right: via surface fitting). Alternative and optional modes in module 1 are indicated on the right sides as dotted boxes. Round boxes indicate the input and outcome of a process, rectangular boxes the processes themselves. Green boxes point to the subsequent processes in the other modules. The 3-d reconstruction **(A)** starts with the input of stereo images, calibration images and technical specifications. Outputs are rectified images and disparity maps on the one hand, which serve as the input data for the subsequent segmentation process. On the other hand, the 3-d point cloud data is transferred to the surface modeling process. With the data provided by the 3-d reconstruction the full or partial recognition of leaves is the intention of the image segmentation **(B)**. On the base of segment-specific points clouds leaf surface structures are modeled in the third module **(C)** either using smoothing operations or bipolynomial surface functions. The resulting polygon mesh of the canopy provides a basis for further statistical analysis of particular plant traits like leaf angles or leaf area.

**Figure 3:**
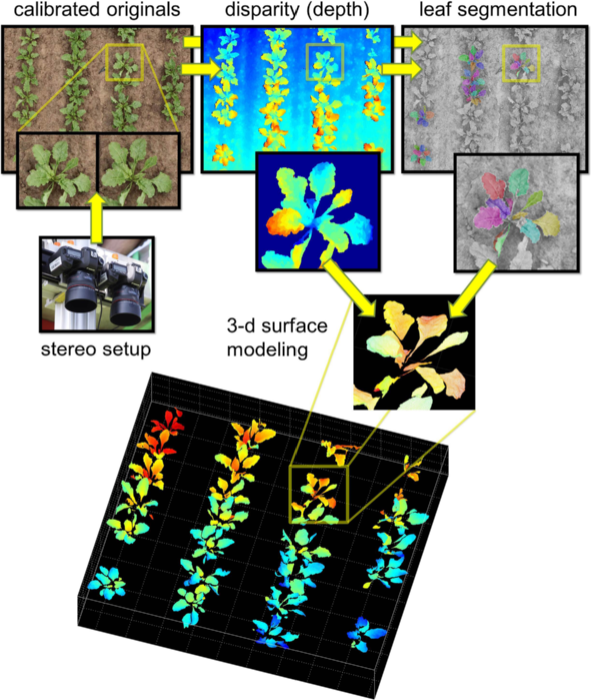
**Example of a complete canopy surface reconstruction.** Illustration of a complete leaf surface modeling process using the example of a young sugar beet population: The modeling starts with the computation of calibrated stereo images taken with an ordinary stereo setup (top left). These images are used in depth estimation (disparity map - top middle) as well as in leaf segmentation (top right). Jet coloration in the disparity map encodes the distance from the camera with blue values being the farthest. The slight off-nadir position of the stereo rig is reflected in the smooth transition of ground values. The enlarged detail displays the image after post-processing, i.e. filtering of the background, occlusions and outliers. Identification of leaf-specific pixels is illustrated in the segmentation image (top right - color-indexed leaves). Results are used for leaf-specific surface modeling (bottom - in this example surfaces have been reconstructed with curvature flow smoothing). After correcting the off-nadir position all surfaces yield the complete 3-d canopy surface reconstruction.

**Figure 4:**
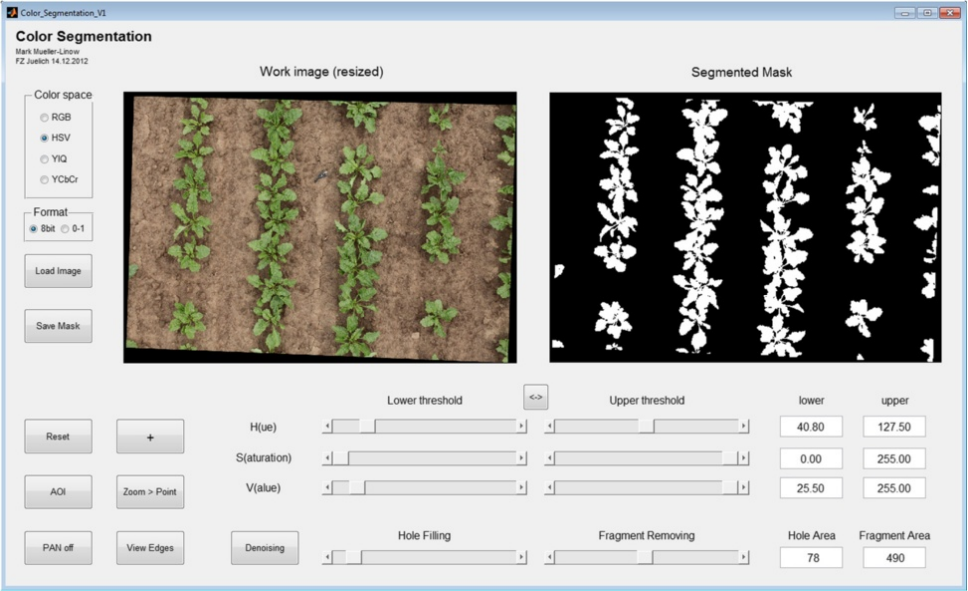
**GUI for background segmentation.** Graphical user interface for background segmentation: RGB image (left) and resulting binary images (right) are applied in 3-d reconstruction (module 1) and leaf segmentation (module 2). Background segmentation is performed in the HSV color space and includes filters for undersized fragments and for the completion of imperfectly filled segments.

Our software package has been developed with Matlab R2012b on a Windows-based platform. Three external toolboxes for image calibration [31-33] and a tool for unstructured 2-d triangular surface meshing [34] are included in the package. Image, calibration and subsequent computed data are organized within a project structure, which builds on a fixed stereo setup. The software consists of 3 essential modules, which control the 3-d reconstruction (i), the leaf segmentation (ii) and the surface modeling (iii), all of them featuring individual graphical user interfaces (GUIs). An outline of each module is given in the block diagram in Figure 2. All modules are interlinked, i.e. some work only with the particular input data (B-C top: green-framed boxes), which come from the first two modules (4 green boxes in Figure 2). Alternative processing options are indicated as dotted boxes, which may be applied. A more vivid view on the overall process is depicted in Figure 3 which uses an example of our case study. The outcome is a 3-d polygon mesh on the base of fitted (planar, quadratic and cubic surface function) or smoothed (Laplacian or curvature flow) leaf surface models, which then can be used for further surface statistics, e.g. estimation of the leaf angle distribution and leaf area index. In addition to surface reconstruction this tool also provides linear, quadratic and cubic modeling of leaf axes and calculation of the respective leaf axes angles. We tested this to be useful for modeling grass-like species, but do not go into details here. We included four additional tools each equipped with a GUI, which will also be outlined here only briefly. The first one (depicted in Figure 4) uses a manual segmentation approach to separate plant pixels from the background. This segmentation, which helps to improve the result in module (i) and (ii), is performed in the HSV color space [35]. The second supplementary tool helps to select the right settings for individual cameras and stereo rig. The the third tool is a visualization tool, which displays the highlights of each processed part together with a summary on settings and estimated parameters, like average leaf inclination and leaf area index. We are also providing an additional tool (with a GUI), which allows for an easy manual post-editing of prior segmentations. The main output of the complete processing pipeline is a surface mesh data file in the well-established ply-format and the leaf angle statistics as an excel-file.

**Figure 5:**
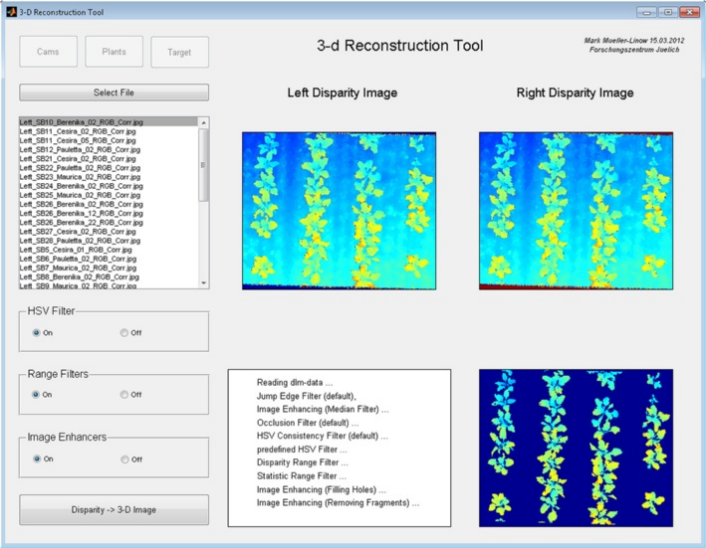
**GUI for 3-d reconstruction.** Graphical user interface for 3-d reconstruction: Several processes are managed with this graphical user interface (module 1), starting with the calibration of the stereo setup, the (auto)calibrated rectification, the 3-d reconstruction of stereo images and ending with several post-processing steps. The current state in the image processing pipeline displays the results of two 3-d reconstructions (top), executed separately for each of the stereo images and the post-processed disparity image (bottom right) after application of all filter options (as listed on the bottom left).

#### Module 1: 3-d reconstruction

**Background** Figure 2A and Figure 5 display the outline and the GUI of the 3-d reconstruction process. Our method of a 3-d reconstruction via the stereo imaging is based on the detection of analogous image information around corresponding points in left (reference) and right (matching) stereo images. We implemented a target-calibrated and uncalibrated (also referred to as auto-calibrated) process both aiming at transformations of the stereo images, which convert all epipolar lines into horizontals (image rectification [36,37]). The target calibration estimates camera geometries (intrinsics) and the geometry of the stereo rig (extrinsics) using a regular calibration pattern [31,38,39]. The auto-calibration mode works solely image-based [32]. Provided that enough and fairly distributed points are detected in both images this method obtains similar results as the target calibration. In the resulting rectified images the correspondence problem [40,41] is reduced to a 1-dimensional search for correspondences. The relative positions of corresponding points, where point coordinates are given with respect to the principal points of left and right camera, denote the depth information (disparity *D*) of the respective pixels. We implemented a block matching method, which makes use of the properties of the surrounding pixels and which uses a couple of statistical measures [40,41], e.g. the correlation *C* of grey values, where corresponding pixels are given by the maximum correlation *C*_*max*_ of blocks [42]. The outcome of the correspondence analysis is raw pixel disparities (depth map), which have to be filtered and finally converted to a 3-d metric point cloud in coordinates (*x*,*y*,*z*).

**Calibration & rectification - default:** We developed a robust auto-tracking algorithm to correctly identify and sort the target pattern features, which are then transferred to the calibration toolbox of Bouguet [31]. The toolbox interfaces have been modified to allow smooth integration to our software. Intrinsics and extrinsics of the camera rig are finally used to rectify the stereo images.

**Auto-calibration - optional:** Auto-calibration requires additional information on the cameras’ focal length *f* and the baseline *b* as well as a certain number of pixel coordinates of corresponding points in the left and right input object image. We combined a scale invariant feature tracking (SIFT) method [33,43] for the detection of corresponding pixels and the auto-calibration toolbox of Fusiello [32]. Rectification is also performed according to the Fusiello method. The estimation process has to be applied on each image pair separately. The Fusiello algorithm shows the tendency of slight image rotations from time to time. We automatically correct these rotations in the module 3: surface reconstruction.

**Correspondence analysis:** Depending on the image resolution and the plant’s surface properties the computation of dense depth maps from stereo images using block method statistics can be computationally demanding. We decided to implement a cross-correlation measure based on the Pearson correlation coefficient. We also tested other block statistics like the sum of squared differences (SSD) and the normalized sum of squared differences (NSSD) for comparison and found only slight deviations from the correlation measure. The computation time and the results of the algorithm mainly depend on 3 parameters, which can be tuned to a certain degree without loosing too much depth information. This parameter set includes the region of interest (ROI), the size of the block *B* used to compare left and right image pixels and the range *R* of the horizontal line which is scanned to find the maximum overlap (e.g. highest correlation *C*_*max*_) of both blocks. In the default mode *B* and *R* are calculated from the camera calibration information together with user-provided plant parameters. So far we examined the two plant species sugar beet and barley, for which standard parameter sets are included in the program. The ROI is defined by the user in the left reference image. The position and range of the corresponding block in the matching image is computed using SIFT-detected [33,43] plant-specific pixels pairs and their relative positions. Another default feature is the increase of depth estimation accuracy beyond the discrete pixel size using sub-pixel fitting [44]. To achieve this the statistical data around *C*_*max*_ is analyzed more deeply using a parabola fit on the neighboring values and recomputing *C*_*max*_ according to the maximum of the fitted curve. The selected region in the rectified reference image is now the basis for all follow up computations like the depth map estimation and the image segmentation. Additionally the correspondence analysis is performed as a two-step process producing two disparity maps. In the first step the left image serves as the reference for the detection of corresponding points in the right one, while in the second step this process is performed the other way around. This additional map is later used when excluding wrong disparity estimations due to occlusions.

**Post-processing & triangulation:** Four default and six optional filters have been included for post-processing: The first two default filter deal with wrong disparity estimations close to leaf edges due to occlusions or strong disparity discontinuities, so-called jump edges. The occlusion filter [45,46] detects inconsistencies between the disparity maps of the correspondence pair analysis and removes the respective pixels. The jump edge filter [47,48] considers the metric distance between a pixel and the 8 neighboring pixels (in the disparity map) for the detection and exclusion of jump edges. The third default filter compares the HSV data of corresponding pixels and removes those with a high discrepancy. We observed this filter to be efficient in suppressing fattening of edges. The fourth default filter is a median filter with a box size of 3 ×3 pixels [49]. The first optional filter analyzes the distribution of disparity values and removes disparities out of range, the second optional filter removes pixel with low statistical significance (i.e. correlation values below a certain threshold). The third optional filter performs a HSV segmentation to separate the plants from the background either automatically or with a pre-defined binary image mask (computed using the GUI in Figure 4). The other three optional filters correct for (i) outliers with a weighted median filter [50], (ii) missing pixels via filling of small gaps, (iii) undersized disparity fragments by removing them. The depth map is finally converted into a 3-d point cloud via triangulation [42].

#### Module 2: Leaf segmentation

**Figure 6:**
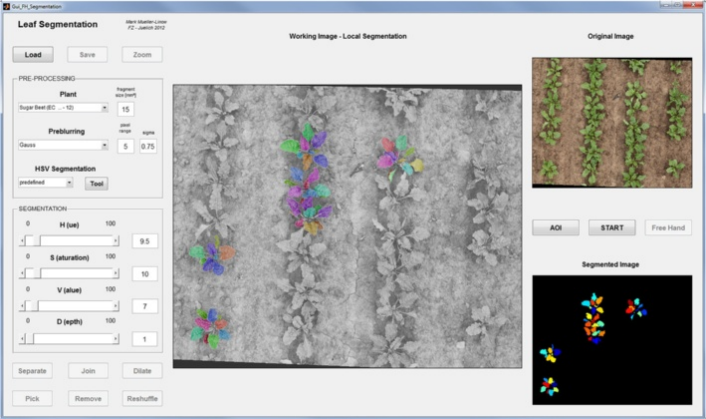
**GUI for leaf segmentation.** Graphical user interface for leaf segmentation: This GUI from module 2 provides a half-automated graph-based method (FH-algorithm) to segment leaves or leaf sections in RGB images. Selection of small interest regions within the input RGB image (top right) allows for a fast FH segmentation with a subsequent detailed editing (with the tools on the bottom left). Segments of acceptable quality may then be transferred to the final output (bottom right). FH segmentation parameters are regulated in the HSV color space together with a threshold for depth separation (left). Pre-processing specifications (e.g. smoothing and background segmentation) are inserted on the top left side. The current state shows an intermediate result of a segmentation for complete leaves.

**Background** Figure 2B and Figure 6 display the outline and the GUI of the leaf segmentation process. To calculate leaf angle distribution each pixel has to be associated with a single leaf and then pixels have to be fitted by a realistic 3-d leaf model. For the planar leaves of soy bean, leaf segmentation was implemented as a graph partitioning method [27]. This method, also referred to as the Felzenszwalb-Huttenlocher (FH) algorithm [51], applies a graph structure on any pre-processed (usually blurred) image information considering pixels as nodes and differences in pixel properties as weighted edges. In the HSV color space, the pixel properties are hue (H), saturation (S) and value (V). We optimized this approach for a better identification of single leaves in various plant species and canopies, as described below.

**Pre-processing:** For blurring we included a Gauss kernel (*σ*_*G*_=0.75) of 5×5 pixel as the default setting, which works well in most situations. For further user-driven optimization optional filters are available (such as Wiener or averaging filters) as well as pre-defined background segmentation (see HSV segmentation in Figure 4). The removal of non-plant image pixels facilitates the manual post-editing as well as the correct separation for dark image regions.

**FH-Algorithm:** As any image information may be used for segment computation we combined the three HSV channels and the disparity values in this GUI, each image property equipped with its own graph partitioning threshold (see Figure 6 left). Single channels or any combination of channels may be used in the FH computation, as from every channel an individual segmentation is calculated, assigning a single label to each segment. The resulting segmentations are then combined to a new segmentation, such that a new segment contains a single label from each channel, only.

**Manual Editing:** Particularly with regard to a more complex leaf surface structure (e.g. sugar beet leaves) the raw segmentation results of each ROI are often not satisfying. We included different manual editing modes, which can be used to achieve a correct segmentation from the pre-segmented image. Over-segmented regions (a leaf consists of several segments) can easily be joined, under-segmented regions (two or more leaves share one segment) can be manually partitioned. In most cases only small junctions have to be cut in order to separate two segments. In the case of strong fragmentations segments may be joined filling the interspaces at the same time. Disconnected segments, which occur due to other overlapping leaves, may also be joined to improve the accuracy of the subsequent surface modeling. An additional free-hand mode may be used for segment completion or dissection. Correct segments are exported to a live-view control image, which is finally post-processed enhancing the segmentation quality (e.g. by filling holes).

#### Module 3: Surface modeling

**Background** We implemented a surface smoothing and a surface fitting approach to model leaf angle distribution. A schematic view on both alternatives is depicted in Figure 2C. Surface smoothing (left) applies curvature flow smoothing [52] or Laplacian smoothing [53,54] to a meshed (via e.g. Delaunay triangulation [55,56] or unstructured triangular surface meshing [34]) set of points to approximate the segment surface. Surface fitting (right) employs different leaf models (3-d surface functions *z*(*x*,*y*)) for the point cloud fitting process via singular value decomposition (SVD) [57] inside a RANSAC loop [58].

**Figure 7:**
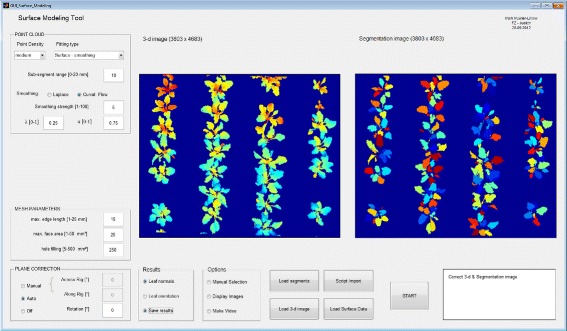
**GUI for surface modeling.** Graphical user interface for surface modeling: Disparity data (left) and segmentation data (right) are combined in module 3 to approximate surfaces to 3-d point clouds according to different modeling options. Surfaces are either fitted according to planar, quadratic or cubic functions or smoothed using curvature flow or Laplace smoothing.

**Surface smoothing:** For pre-processing we included a filter, which removes disjoined point clusters up to a particular size and distance to the major clusters. The resulting point clouds are firstly meshed via 2-d Delaunay triangulation on the base of *x* and *y*-coordinates to ensure the formation of only such faces reflecting the leaf surface. Faces with edge lengths or areas above predefined thresholds are removed in order to prevent cross-linking in non-plant sections (Figure 7 left - mesh parameters). The final 3-d triangulation, which is performed via unstructured triangular surface meshing, follows on the identification of the leaf border and holes. Holes within each segment, which appear due to fragmentary disparity maps and missing segmentation information, are identified and automatically removed, if they are below a predefined size. Resulting segment meshes are then individually smoothed with the curvature flow or the Laplace method depending on the user’s choice. Curvature flow requires 3 parameters, which are preset by the user and which regulate smoothing iteration, smoothing strength and neighborhood impact (Figure 7 left - point cloud).

**Surface fitting:** Surface fitting aims at adapting coefficients *c* of a user-selected leaf surface model *z*_*c*_(*x*,*y*) to a point cloud segment, in order to compute leaf angle and curvature. It joins two processes: Finding a consensus set of points in the given point cloud segment via RANSAC thereby deleting outliers and the estimation of surface model coefficients *c* via singular value decomposition (SVD). The amount of points in the consensus set strongly depends on the number of iterations used to determine the surface coefficients in randomly composed subsets. This number is proportional to the ratio of segment size to subset size. A distance criterion is used to determine whether a subset contributes to the consensus set or not. The surface coefficients, which are then computed from the consensus set via SVD, determine the curvature of the segment. Meshing steps run analogous to the path described in the last section.

**Post-processing:** For further computations (see case study below) it is important to adjust the final outcome with respect to the inclination and orientation of the stereo rig. The latter one can be achieved by rotating the surface points around the *z*-axis, while the first one needs a transformation of the *xy*-plane (Figure 7 left - plane correction). We included two options to correct the data: (i) The user may record and provide the inclination and direction of the stereo rig manually (with the help of an inclinometer and a compass) or (ii) he may use the automatic mode. Assuming that plants have been grown homogeneously in a near-flat soil, a plane will be fitted through the plant pixel coordinates and used for the computation of the plot inclination.

### Leaf surface traits

**Leaf area and leaf area index:** The software provides a leaf-specific area estimation and the computation of the leaf area index (LAI) by relating the estimated total leaf area to the area covered by the selected image region. In sparse canopies, where leaf occlusion is neglectable these values can be taken as face values. In denser canopies with occlusion and leaf clumping the visible proportion of leaves has to be extrapolated to total leaf area, which requires assumptions on the non-visual part of the canopy. Various approaches are available in the literature and the output of our program can be used for these forward calculations [59,60]. Because of the large number of possible approaches that are developed for the various canopies, we did not implement any method in our software.

**Leaf angle distribution:** This software tools allow for a statistical assessment of leaf orientations of single plants and small plant populations with a representative number of individuals. A mathematical equivalent for leaf orientation is the leaf angle or leaf angle distribution. Our methods and algorithms are designed for the local derivation of leaf angles on the base of individual leaf models. The leaf angle distribution summarizes the properties of individual leaf surface models. The orientation of each face within such a Delaunay-triangulated surface can be expressed by 2 angles in a spherical coordinate system, the azimuth *θ* and zenith *φ*. Typically the zenith angle varies from -90° <*φ*<90°. In our convention we assume that each face normal and the normal of the soil span an angle of less than 90°, meaning face normals point ‘up’. Therefore the zenith angle will only range from 0<*φ*<90°, with a value of *φ*= 0° reflecting a horizontal face. Azimuth angles range from 0° <*θ*<360° starting at the right-hand side in the image and then turning counterclockwise.

### Error estimation

**Figure 8:**
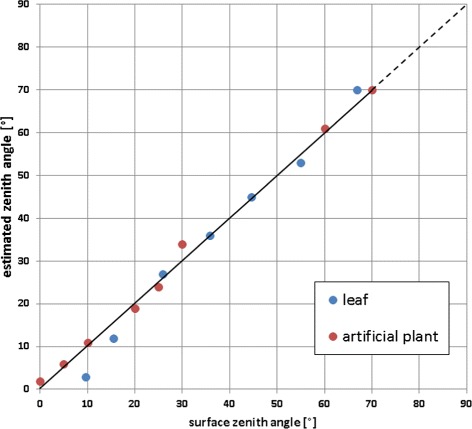
**Error estimation.** Surface zenith angles of artificial setups were measured with an inclinometer and compared to respective estimated leaf angles from stereo reconstruction. Estimation errors were quantified by the normalized root mean square error (NRMSE): artificial plant leaves (red) displayed a NRMSE of 2.5%; planarly-fixed sugar beet leaf in various orientations (blue) displayed a NRMSE of 4.6%. The dashed line indicates where angle estimates are known to be unreliable [27].

We tested the accuracy of leaf angle estimation in two experiments. The first one uses an artificial plant with 8 green-colored flat leaves made form plywood which can be adjusted to any zenith angle. The second one employs a sugar beet leaf fixed on a flat surface, which could be oriented arbitrarily. Targets were imaged from nadir position (3.5 m distance) with two Canon EOS 5D Mark II (*f*=50 mm; *b*≈200 mm). We set the leaves of the artificial plant target to different zenith angles, such that most parts of each leaf were in camera view. Individual leaf angles were manually measured using a high resolution dual-axis digital inclinometer (Level Developments LD-2M). Inclination of the sugar beet leaf was manually changed and measured between each imaging step. Here we applied 7 different orientations. Images were processed using the target calibration pipeline. Leaves were segmented and fitted with a planar surface model. Figure 8 summarizes results for both tests. The deviation from the identity line was quantified for the accessible zenith angle interval [0°, 70°]. Steeper leaf parts are not well visible and thus do not give reliable angle estimates [27]. The normalized root mean square error (NRMSE) is approx. 2.5% for the artificial plant and approx. 4.6% for the fixed leaf. Moreover, we computed the sugar beet leaf area of all orientations and estimated the error using the normalized variation coefficient, which is approx. 2.8%. We do not observe a bias towards fronto-parallel surfaces well known for other stereo reconstruction approaches [61].

## Case study

**Figure 9:**
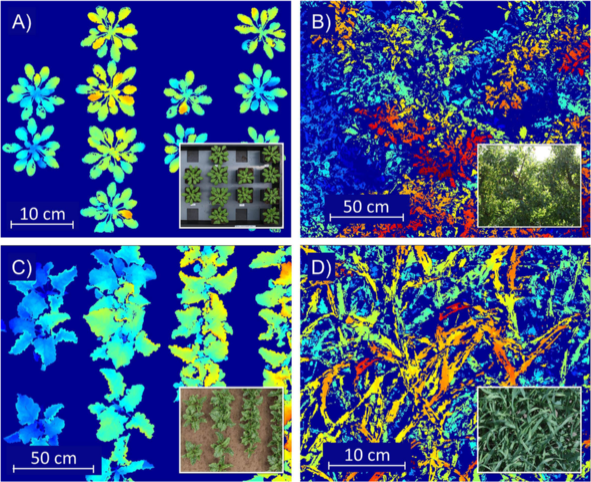
**Field of applications.** Depth maps and respective RGBs (inlay) of different experimental plant systems: Pixel disparities in the depth map are color-coded ranging from red (closer to cameras) to blue (further away); distance ranges are given in brackets: **(A)** trays of *Arabidopsis thaliana* were monitored in studies of diurnal leaf movement (≈20 *m* *m*); **(B)** single trees (apple orchards) were analyzed with respect to the leaf and fruit stratification; Klein-Altendorf, 2013 (≈700 *m* *m*); **(C)** small plot of sugar beet; case study from CROP.SENSe.net central experiment (Campus Klein-Altendorf); 2012, June 14 (≈500 *m* *m*); **(D)** small barley populations; Crop Garden experiment at FZ Juelich; 2011, July 5 (≈400 *m* *m*).

Our stereo imaging approach has been tested with different plants demonstrating the functionality of our software across species and applications (Figure 9). Our test cases ranged from the small rosette plant *Arabidopsis thaliana* (Figure 9A) to single trees in apple orchards (Figure 9B) to the agricultural crops sugar beet and barley, which are the main focus species of the Crop.Sense.net network (Figure 9C, D). Currently further studies with *Arabidopsis* are on the way to better understand gene-phenotype interactions and with apple trees to assess fruit traits by 3-d stereo imaging (results will be published elsewhere). In this manuscript we focus on a detailed investigation of four different sugar beet varieties that were subjected to different nitrogen availability. We performed a detailed case study demonstrating the potential of our stereo approach to distinguish subtle seasonal, variety and treatment specific differences in leaf display.

**Plants** The cultivars *Cesira*, *Pauletta*, *Maurica* and *Berenika* have been grown in 6 × 20 meter plots with 4 repetitions in the central experiment of CROP.SENSe.net in the agricultural study site of Campus Klein-Altendorf (NRW, Germany). Two nitrogen treatments (80 kg/ha and 150 kg/ha) have been applied (in the following denoted as *N*- and *N*+). The cultivar and treatment combinations each with 4 repetitions were planted in plots of 6 × 20 m.

**Measurements** We used two Canon EOS 5D Mark II with *f*=50 mm lenses mounted on a sliding bar with a baseline of *b*≈200 mm (Figure 1). The stereo rig was mounted on a bucket truck for imaging at nadir position 3.5 m above the canopy. The remote-control release for simultaneous triggering consisted of two pre-trigger remote cables fixed to one trigger button. Camera calibrations were performed as usual with regular 2-d pattern. For our purposes we designed a rectangular frame with a square-like alignment of 9 × 6 black circles in front of a white background. The pattern was attached to a flat surface. Calibration quality depends on the number of different target positions, which can be achieved by rotating the calibration pattern. We took at least 20 images covering the whole plant volume. The target imaging distance was similar to the imaging distance to the canopy. The three measurement days in 2012 were May 30 (*s*), June 14 (*m*) and September 5 (*l*). 2 repetitions of each cultivar & treatment combination were imaged at a time resulting in a total of 48 stereo images. These dates correspond to different soil coverage ranging from minor coverage (as depicted in Figure 4) up to complete soil coverage. Images were taken with the same platform orientation around noon (±2*h*). Distance to the canopy was fixed with a laser range finder (Bosch PLR 25) and inclination of the platform was monitored with a high resolution dual-axis digital inclinometer (Level Developments LD-2M). As the cherrypicker platform was always facing east, no further viewing direction data for stereo rig correction has been recorded.

### Image processing setup

Images were processed starting with the default calibration pipeline. Figure 3 displays exemplarily the overall surface reconstruction of a sugar beet plant population in an early state (*Berenika* - May 30). The reconstruction worked well for all images, however problems occurred at strongly reflecting leaf surfaces. These parts tend to display hot spots, which move with the viewpoint (non-Lambertian surface effect) leading to wrong disparity estimations especially along the midribs that are orientated parallel to the stereo rig axis. These sections are mostly excluded by the filtering processes. In the following, we tested both surface modeling modes (smoothing and fitting) with regard to the plausibility of results. For surface smoothing we used segmented point clouds of entire leaves. Leaf segments were pre-filtered separately eliminating point clusters with spatial separation of >5 mm and resulting meshes were smoothed using curvature flow. Surface fitting was performed on leaf segments cut at the midrib. This additional step is necessary as sugar beet leaves tend to be folded along the longitudinal leaf axis depending on variety and stage. The resulting segmented point cloud was then fitted with a quadratic surface function inside a RANSAC loop using subsets of 50 points and a distance criterion of 1.75 mm. In both approaches the orientation of the surface meshes was finally corrected with the recorded inclination data of the stereo rig position. We also checked the automatic correction mode, which produced similar results. Both methods were in principle capable of capturing essential leaf properties which reflect seasonal and genetic differences, however the smoothed leaf model results displayed a higher visual plausibility, which is why we will focus on these in the following.

### Estimation of the leaf angle distribution

**Figure 10:**
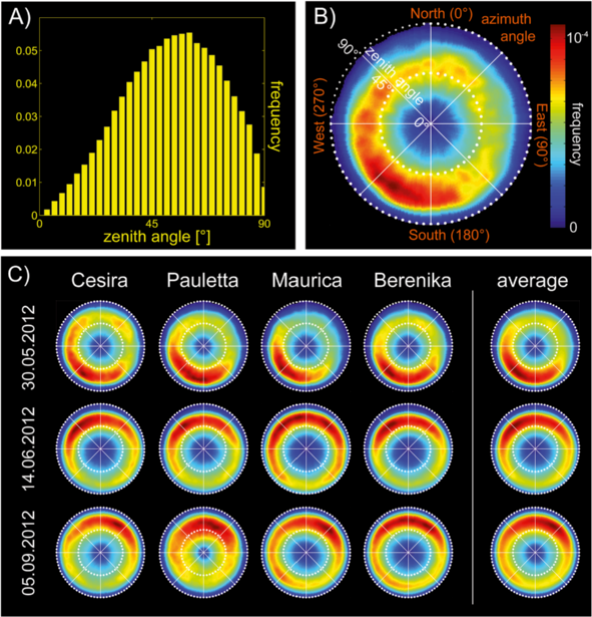
**Leaf angle distribution of a sugar beet sample.** Leaf angle distributions of sugar beet populations: **(A)** frequency of zenith angles; **(B)** combined zenith and azimuth angles in a 2-d histogram plot for a single image of *Berenika* (May 30/ *N*+), the example given in Figure 3. Curvature flow smoothing has been applied on the 3-d data. In this representation the azimuth angles *θ*= 0°, 90°, 180° and 270° correspond to the cardinal points North, East, South and West. **(C)** compares state effects (date of measurement) and cultivar effects, each diagram representing the average of 2 repetitions and 2 different nitrogen treatments: As indicated by the overall averages (right), the growth state differences are most obviously reflected in the location and center of the azimuth distribution. (All images have been generated with an extra visualization tool).

The orientation of each face (as given by the face normal) within the reconstructed meshes can easily be converted into azimuth (*θ*) and zenith (*φ*) angles and - if related to the individual face area - summed up to the distribution of leaf angles. Distributions are calculated as normalized histograms with 1° bin width, if not stated differently. The interpretation may be carried out on the basis of *φ* or *θ* angle distributions separately or as joint distributions, assuming that there is a dependency between both distributions. (i) Figure 10A shows the distribution of *φ* for *Berenika* (May 30/ *N*+). Further statistics measures like the mean or median of the *φ* distribution may help to characterize drought stress, diseases or diurnal leaf movements. The shape of the *φ* distribution displayed characteristic differences between the cultivars, which were distinctly increased in the older developmental states, while for the *θ* angle distributions we recorded seasonal differences particularly reflected in the location of the angle averages (data not shown). (ii) On a higher level *θ* and *φ* distributions are combined in a two-dimensional histogram with the angular axis denoting *θ* and the radial axis denoting *φ* (Figure 10B). The *θ* distribution is not uniform and there are clusters of increased frequency, in this representation in the southwestern hemisphere with a maximum around *θ*= 220°. This in turn means that a comparatively large amount of leaf parts are facing Southwest. In the following we checked the consistency of results with respect to genetic and environmental conditions. The setup of the Central Experiment of Campus Klein-Altendorf displays four major factors with potential influence on the phenotype, namely state, genotypic, site, and fertilization effects. Most obvious are the strong morphological changes of all sugar beet cultivars during seasonal development (state effects), while variety-specific differences become more apparent the older the leaves are (genotypic effects). We expected less impact from site (repetitions) and from fertilization (nitrogen treatment) effects, which is why we averaged over these two factors in Figure 10C, displaying the distributions of all state-cultivar combinations. Looking at the state effects one observes a cultivar-independent preferred direction in *θ* (also illustrated by the averages on the right) which changes distinctly over the season especially from the youngest to the older states. The center of the azimuth distribution, which is the median $\tilde {\theta }$ of the distribution, is shifted from Southwest ($\tilde {\theta _{s}}=$ 217°) to North ($\tilde {\theta _{m}}=$ 354° and $\tilde {\theta _{l}}=$ 4°). We do not know yet the exact cause for this effect, but there are several reasons, that have to be taken into account: (i) Strong west winds at the location of our study site at Campus Klein-Altendorf may affect the leaf orientation and plants may react to windy conditions differently according to their susceptibility (leaf size or closeness of the canopy due to the sawing pattern); (ii) sugar beets are sown in rows from east to west with a row distance of 50 cm and a plant distance of 20 cm within the rows. Plants in the youngest state *s* do not have any contact to their neighbors. Plants in the intermediate state *m* have contact to the plants within their rows, plants in the oldest state *l* also across the rows. Therefore, young plants with small leaves and no neighborhood competition may orientate their leaves most efficiently to the south, while in older plants this deviation from the optimal distribution may be caused by a combination of several factors. Concerning the distribution of *φ* the results are rather homogeneous, however differences between states and cultivars are present, particularly in the shape of the distributions. The width of the *φ* distribution for instance differs strongly between *Pauletta* and *Berenika* in all states being much broader in the case of *Pauletta* and also the youngest states display lower average *φ* angles with medians of $\tilde {\varphi _{s}}=49\pm $1° compared to the older states with $\tilde {\varphi _{m}}=56\,\pm $ 2° and $\tilde {\varphi _{l}}=53\,\pm $ 5°. Again, this finding may result from a combination of different effects: (i) leaf stages differ distinctly between plants at younger or older states and (ii) leaf erection as a result of neighborhood interaction. Young plants have enough space to adjust their leaves optimally for light interception, while older plants have to compete for light on the one hand and are featured by very large and heavy leaves with planophile leaf sections on the other.

**Figure 11:**
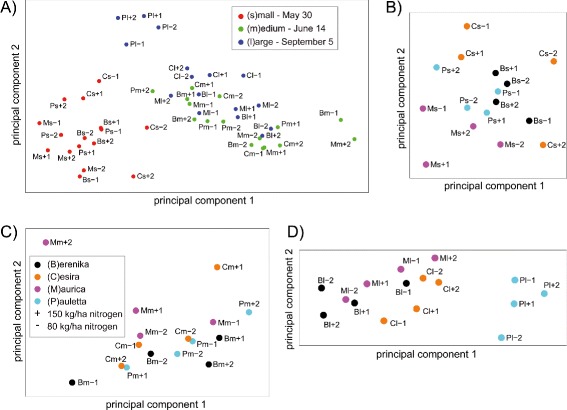
**Principal component analysis (PCA) of plot-specific leaf angle distributions.** PCA reveals distinct clustering of states and cultivars, while nitrogen treatment effects are lacking. **(A)** Complete set of 48 analyzed images representing 2 repetitions (1,2) of 24 parameter constellations - 4 cultivars (B,C,M,P), 3 states (s, m, l) and 2 nitrogen treatments (+, -): The states (color-indexed measurement days) display strong clustering with the strongest separation of the youngest plant state. **(B-D)** Analysis of each state indicates variety-specific leaf angle distributions resulting in a pronounced clustering of all 4 cultivars (color-indexed) regardless of the measurement day.

As these results displayed only slight differences between the cultivars, we analyzed the leaf angle distributions of the 48 combinations (measurement date, cultivar, nitrogen treatment and repetition) more deeply by performing a principle component analysis (PCA). To this end we interpret each angle distribution with its *N* bins as a point in an *N*-dimensional space, i.e. we populate this space with 48 points. PCA then delivers directions of main variations in this *N*-dimensional space. We investigated clustering effects when using *θ*- and *φ*-distributions separately or jointly. As effects were more pronounced for the latter case, we focus on this analysis in the following. The first two components of the PCA have been depicted in Figure 11A. Most apparently, the three plant states are well clustered (as indicated by the three colors) and also separated in the case of the youngest state *s*. There is no systematics within the distribution of nitrogen treatments, but clustering of cultivars is present within each sub-group. For a detailed analysis we repeated the PCA separately for each measurement date (Figure 11B-D). As indicated before, all states are featured by a fairly good separation of cultivars, especially for the last state *l*, while nitrogen treatment effects seem to be negligible. This study was also carried out with a planar leaf model and the quadratic surface function model. The results were comparable but less pronounced than with the model-free surface smoothing option.

## Discussion

The reconstructibility of plant canopies from stereo images captured in the lab or under field conditions strongly depends on hardware setup, imaging conditions, plant and planting type. (i) The spatial relation between cameras and between stereo rig and plants affect the field of view, the spatial accuracy (depth and lateral resolution), the degree of occlusions and the reconstructable depth range due to the depth of focus. In the following, we name a few parameters, which should be handled in a balanced manner (our additional stereo rig setup tool is intended for this purpose): Increasing baseline *b* results in a better depth resolution but produces more occlusions. Using lenses with a higher focal length *f* also increases the depth resolution, but decreases the field of view, which is also decreased by smaller distances between stereo rig and canopy, while the depth resolution is increased. A higher F-number (aperture) gives a better depth of focus, but also increases the exposure time, which can cause problems in a shifting canopy. (ii) Alternating light conditions and heterogeneous illumination across the canopy are sources of erroneous depth estimations and may cause problems in automatized segmentation processes. Strong incident solar radiation together with specular properties of the leaf surface often produce hot spots and a decrease of visible texture and should therefore be avoided, e.g. by measuring under diffuse light conditions (clouded sky). Windy conditions during image acquisition strongly affect the quality of reconstruction and should also be avoided. Short exposure times are required in such cases. (iii) The type of plant, plant age and planting density strongly affect the results. Taking the camera perspective into account the degree of self and of mutual occlusion (leaf clumping) defines the proportion of reconstructable plant material. The amount of image pixels covering the narrow side of the leaf blade should be large enough for a correct surface reconstruction (e.g. barley leaves should be imaged from a shorter distance or with a higher *f*). Plant leaves with homogeneous surface properties (e.g. barley) may cause wrong disparity values. This effect is particularly strong for specular reflections of sun light, where the hot spots move with the camera perspective, leading to wrong assignments of corresponding image regions. iv) Calibration issues may play a role when the camera system is not used in a fixed setup, which needs to be calibrated only once. If used more flexible, the setup needs to be re-calibrated after every change. If deficient rectifications occur, images can still be analyzed using the auto-calibration mode. v) For the surface fitting process the right choice between smoothing or surface functions strongly depends on the leaf surface properties and on the amount of noise in the data and should therefore be taken accordingly. Approximating the leaf surface from smoothed data might be appropriate for complex leaf geometries, for low-noise data sets and for studies, where a leaf angle resolution on the pixel-level is required. For the estimation of a single leaf-specific angle or for noisy data surface model fitting should be preferred.

## Conclusions

With the development of this software package we want to provide a comprehensive tool for the analysis of leaf surface properties within the outer canopy layers using off-the-shelf hardware, which can easily be assembled to a stereo camera rig. With little methodic effort, the generation of dense depth maps, the identification of single leaves and the modeling of the leaf surface structure is feasible. The automatic segmentation of single leaves is possible, however for dense canopies (e.g. older sugar beet plants in the stock) leaf segmentation requires some manual editing despite the information available on the vertical leaf distribution. This is a step, where user interaction is required, and we think that it can be reduced to a minimum with plants that have a more homogeneous shape and curvature. Surface modeling is the delicate step in the processing pipeline and the method of choice strongly depends on the quality of data, the complexity of leaf structure and the scientific questions. Despite the methodic restriction of the reconstructibility to the outer canopy layers, these observations can be of great use as these layers are of utmost importance for photosynthetic activity. Therefore shifting states in that part may serve as a proxy for the physiological and the health status, respectively. Employing the properties of the outer canopy typical seasonal and genotypic differences between our plant populations were clearly demonstrated and we think that this method is applicable to other fields up to the level of agricultural and horticultural plant systems.

## Availability and requirements

**Project name:** Leaf Angle Distribution Toolbox**Operating system:** Windows**Programming language:** Matlab**Other requirements:** Matlab Compiler Runtime (MCR)**License:** proprietary - the software is restricted to academic use only. The software is available from the authors upon request. Research projects, which benefit from the Leaf Angle Distribution Toolbox, are obliged to cite this paper.

## Abbreviations

*b*: baseline, distance between the camera centers
*B*: size of the block used in the block matching method of the correspondence analysis
*R*: search range for corresponding points in the correspondence analysis
*f* or *f*_*p*_: focal length [mm / pixel]
*D*_*ij*_: disparity of pixel in column *i* and row *j*
*x*_*ij*_, *y*_*ij*_, *z*_*ij*_: 3-d coordinates of a pixel in column *i* and row *j*
*σ*_*G*_: Gaussian kernel segmentation
*N*- / *N*+: nitrogen treatment (80 kg/ha or 150 kg/ha)
*θ*: azimuth
*φ*: zenith
*s*, *m*, *l*: plant states: small (30.05.2012), medium (14.06.2012), large (05.09.2012)
